# Lumbar Facet Joint Radiofrequency Ablation With a 3-Tined Cannula: A Technical Report and Observational Study

**DOI:** 10.1155/prm/8871568

**Published:** 2024-12-20

**Authors:** Andrea Künzle, Sander M. J. van Kuijk, Eva Koetsier

**Affiliations:** ^1^Faculty of Biomedical Sciences, Università della Svizzera Italiana, Lugano, Switzerland; ^2^Pain Management Center, Neurocenter of Southern Switzerland, EOC, Lugano, Switzerland; ^3^Department of Clinical Epidemiology and Medical Technology Assessment, Maastricht University Medical Center+, Maastricht, Netherlands

**Keywords:** ablation, denervation, facet joint pain, low back pain, lumbar, multitined cannula, observational study, perpendicular approach, radiofrequency

## Abstract

**Background:** Lumbar facet joints are the source of pain in 15%–41% of individuals experiencing low back pain (LBP). Conventional lumbar facet radiofrequency ablation (RFA) has Level II evidence for improving pain and function. The best proven technique, the parallel technique, is technically challenging, time-consuming, and often uncomfortable for the patient. A novel RFA technique using a 3-tined cannula offers a potentially less complex and shorter procedure.

**Objectives:** To describe the novel lumbar facet joint RFA technique with the 3-tined cannula and to evaluate its efficacy in treating chronic lumbar facet joint pain.

**Methods:** Eligible adult patients with chronic lumbar facet joint pain, confirmed by positive medial branch blocks (MBBs), refractory to conservative treatment, received the novel RFA treatment with the 3-tined cannula. The change in pain intensity at 2 months follow-up compared to baseline, percentage of patients reporting a ≥ 30% and ≥ 50% reduction of pain intensity, patient global impression of change (PGIC), need for pain medication, walking ability, sleep quality, and patient satisfaction were evaluated.

**Results:** A total of 44 patients were included. Patients experienced a clinically meaningful and significant pain relief at follow-up and 41% of the patients reported ≥ 50% reduction of pain. Forty-eight percent experienced at least a score of “much improved” on the PGIC. No severe side effects or complications were observed.

**Conclusions:** Our observational study suggests that lumbar facet joint RFA using the novel technique achieves significant pain relief. The larger lesions decrease the likelihood of missing the target nerve while obviating the need to conduct numerous lesions. Limiting is the single-center set-up with a relatively short-term follow-up duration. Randomized controlled clinical trials are warranted to confirm the efficacy of the novel RFA technique to treat lumbar facet joint pain.

## 1. Introduction

The lumbar facet joints, also known as zygapophyseal joints, have been implicated as the source of pain in 15%–41% of all patients with low back pain (LBP) [1, 2]. Patients typically complain of paramedian axial LBP, often irradiating to the buttocks, groin, or thigh [3–5]. Diagnostic local anesthetic nerve blocks of the medial branches of the dorsal ramus of the lumbar spinal nerves are a reliable method to establish a diagnosis of facetogenic LBP, as these nerves provide sensory innervation from the facet joints [1, 6, 7]. Guidelines recommend that conservative treatment (e.g., nonsteroidal anti-inflammatory drugs [NSAIDs], physical therapy, and LBP education) should be attempted for at least 3 months before considering proceeding with medial branch blocks (MBBs) [8, 9].

**Figure 1:**
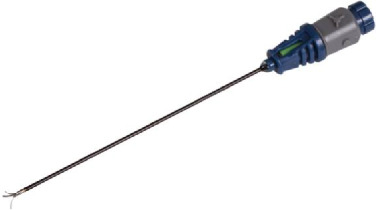
18-gauge 3-tined RFA cannula with a 5-mm active tip with the 3 tines deployed, separated by 120°. Tine deployment is achieved by rotation of the gray collar on the hub of the cannula. RFA = radiofrequency ablation.

MBBs providing pain relief for the expected duration of action of the local anesthetic select patients for radiofrequency ablation (RFA) [10]. RFA delivers an electrical current that passes in close proximity to the target nerve, resulting in heat production leading to the coagulation of the nerve [11, 12]. Lumbar facet RFA with a monopolar cannula has Level II (moderate) evidence supporting its effectiveness in improving pain and function [13–15]. The most recommended and best proven technique is the parallel technique [8, 11] positioning the cannula parallel to the medial branch nerves [8, 11, 16–18]. Multiple parallel cannula placements are recommended to increase the likelihood of capturing the nerve and successfully ablating the target nerve [19]. However, this technique is technically challenging, time-consuming, and often uncomfortable for the patient. Recently, a novel 3-tined cannula with an increased active area has been manufactured (Figure 1), allowing for a RFA technique with a perpendicular approach that requires only a single cannula placement [20]. The novel RFA technique offers a potentially less complex and shorter procedure than the conventional lumbar facet joint RFA.

The objective of this study was to describe the novel lumbar facet joint RFA technique with the 3-tined cannula and to evaluate its efficacy in treating chronic lumbar facet joint pain.

## 2. Methods

Permission to analyze data from the prospectively maintained cohort of patients treated with the novel RFA procedure in our interventional pain management center was obtained from the Research Ethics Committee (CE 4024), Switzerland. All patients provided written informed consent. The trial was registered on ClinicalTrials.gov.

### 2.1. Patients

Inclusion criteria involved patients of at least 18 years of age with a history of chronic LBP, without a radicular pattern or neurologic symptoms in the lower extremity, persisting for at least 3 months despite conservative treatment (e.g., NSAIDs, exercise regimens, and physical therapy). All patients had to have undergone two MBBs on separate occasions, resulting in at least 50% temporary pain relief. Patients who were pregnant, on anticoagulant medication, had a systemic or local infection, or had a pacemaker were excluded. Patients who underwent more than one RFA treatment, did not provide informed consent, or had incomplete data from pre-RFA measurements or follow-up due to missed appointments were also excluded.

### 2.2. Diagnostic MBBs

Targeted facet joint levels were determined by clinical presentation (e.g., history, paraspinal tenderness, and referred pain pattern), and imaging (MRI). The MBB procedure was performed in a sterile ambulatory setting, with the patient lying prone on the fluoroscopy table and a pillow placed under the lower abdomen to flatten the lumbar lordosis. The C-arm was declined to square off the superior endplate of the targeted vertebra. With this declination, an oblique fluoroscopic view was obtained toward the side of the pathology, revealing the junction between the superior articular process and the transverse process of the targeted vertebra.

The MBB procedures adhered to the International Pain Society and Interventional Spine Society practice guidelines [11]. Dual diagnostic MBBs were conducted with intermittent fluoroscopic visualization utilizing a 25-gauge needle (BBraun: 25G, 88 mm, Spinocan needle, Melsungen AG, Germany). Each facet joint is innervated by two medial branches: one at the adjacent same level as the joint with the suspected pathology and the other at the upper level [21]. Therefore, the MBB was performed at the level of the joint with the suspected pathology and at the level above. The medial branch nerves from L1 to L4 traverse the groove formed by the junction of the superior articular process and the transverse process. The needle is advanced along the direction of the X-ray beam (tunnel vision), perpendicular to the medial branch nerve. The target point for the cannula placement is the midpoint of the root of the dorsal surface of the transverse process (at the eye of the Scottie dog [22]), just inferior to the superior junction of the superior articular process and transverse process of the L1–L5. The L5 target is the L5 dorsal ramus running along the groove between the ala of the sacrum and the superior articular process of S1, instead of the less accessible L5 medial branch nerve. This target point is located just inferior to the superior junction of the S1 superior articular process and the sacral ala. At the aforementioned target points, 0.5 mL of 0.5% bupivacaine was injected.

In the three hours following the MBB procedure, patients were instructed to maintain a pain diary and rated their pain intensity every 30 min using the 11-point numeric rating scale (NRS). The scale is represented as zero for no pain and 10 for the worst pain imaginable [11]. After the completion of the pain diary, the interventional pain physician verbally verified the MBB response with the patient. Diagnostic MBBs resulting in ≥ 50% pain reduction on the 11-point NRS compared to baseline were considered positive blocks [8, 23].

### 2.3. Novel RFA Technique With the 3-Tined RFA Cannula

All RFA procedures were likewise performed under fluoroscopy, with the patient lying prone, with a pillow under the lower abdomen. The needle insertion point was identified on the skin, and at this point, a subcutaneous injection of 1–2 mL of lidocaine 1% was administered using a 25-gauge needle (BBraun: 25G, 40 mm, [1½ inch], orange, sterile hypodermic needle, Melsungen AG, Germany). With the novel RFA technique using the 3-tined needle, the cannula can be positioned perpendicular to the nerve. This approach is similar to the approach used for the lumbar diagnostic MBB. Under fluoroscopic guidance, the 3-tined 18-gauge, 90 mm RFA cannula, made from medical-grade stainless steel and insulated except at its 5 mm active tip (Diros OWL RF Trident Cannula, DTR-018/100/5, Diros Technology Inc. Markham ON, Canada) was steered towards the target point for the MBB and/or the L5 dorsal ramus target. Lateral and posteroanterior fluoroscopic views were taken to confirm that the RFA cannula tip was accurately positioned against the bony pillar surface. Whether or not patients had prior posterior spinal instrumentation did not alter the approach; however, in some cases, we adjusted the C-arm further laterally in the oblique view and used a slightly more lateral insertion point to navigate around existing hardware. In accordance with the Spine Intervention Society Practice Guidelines [11], sensory and motor stimulation were not performed.

**Figure 2:**
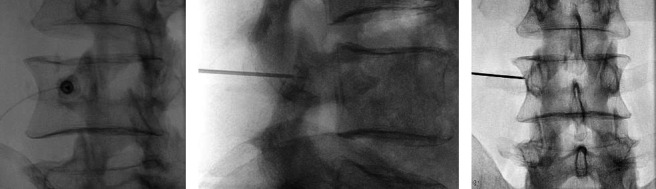
Fluoroscopic image demonstrating the positioning of the cannula with the 3 tines deployed and targeted at the level of the left L3 medial branch nerve. The oblique view (a), the lateral view (b), and anteroposterior view (c) are shown. RFA = radiofrequency ablation.

The medial branch nerve was anesthetized with the injection of 1-2 mL of lidocaine 1% through the RFA cannula. The physician connected the RF cannula to the RF generator (ABBOTT MEDICAL, 6901 Plano Texas 75024, the United States of America), and the grounding pad was placed on the patient's thigh. The interventional pain physician deployed the three tines by rotating the handle (Figures 1 and 2). This rotation gradually reveals a green segment in the viewing window, confirming full tine deployment and alignment, enhancing precision and assurance during placement. After final fluoroscopic control with oblique, posteroanterior, and lateral views, the thermal lesions were delivered at 80°C for 90 s per ablation [24]. The fluoroscopic images of final needle positions are shown in Figure 2.

If the patient reported pain from the heating of the cannula tip, the interventional pain physician temporarily paused the RFA lesioning and injected additional local anesthetic through the RFA cannula. All interventions were performed by one interventional pain physician (EK) with 10 years of experience in performing fluoroscopically guided neuraxial procedures.

### 2.4. Outcome Measures

The primary outcome of this study was the change in pain intensity at the 2-month follow-up compared to baseline. Pain intensity was measured using the 11-point NRS. According to the methods, measurement, and pain assessment in clinical trials (IMMPACT) guidelines [25], a pain reduction of two points was considered as a clinically meaningful benefit. Secondary outcomes included the proportion of patients reporting ≥ 30% and ≥ 50% pain reduction 2 months after RFA. Following IMMPACT recommendations [25], a 30% pain reduction indicates at least a moderate clinically important difference. Additionally, since reductions in chronic pain intensity of 50% indicate substantial improvements, it is also recommended to report the proportion of patients showing this degree of improvement. Additional secondary outcomes included patient global impression of change (PGIC), pain medication use, sleep quality, walking ability, and patient satisfaction at the 2-month follow-up. Demographic and clinical variables collected included age, sex, duration of pain, prior lumbar spine surgery, existing posterior spinal instrumentation at the ablated levels, laterality, and procedure dates. Patients were instructed to contact our clinic if they experienced adverse events or complications following the procedure. A follow-up visit was scheduled for all patients 2 months after the RFA treatment. To prevent bias, individuals responsible for data collection and analysis (Andrea Künzle and Sander M. J. van Kuijk) were not engaged in the execution of the RFA procedures.

### 2.5. Statistical Analysis

The sample size was determined pragmatically by including all eligible patients treated with the 3-tined RFA cannula during the study period. Baseline characteristics were expressed as mean and standard deviation (SD) or as count and percentage. Missing pain intensity scores were imputed using stochastic regression imputation to prevent loss of power and precision and to decrease the risk of selection bias associated with using only complete cases. We used the paired-samples *t*-test to test the difference between mean baseline and post treatment NRS scores. This analysis was repeatedly stratified by the number of facet joints treated. The Type-I error rate was set to 0.05. Treatment success was defined as scoring “minimally improved,” “much improved,” or “very much improved” on the PGIC and was reported as count and percentage. Other secondary outcomes which assessed only posttreatment were reported as count and percentage. The analyses were performed in Microsoft Excel and R version 4.0.4.

## 3. Results

**Figure 3:**
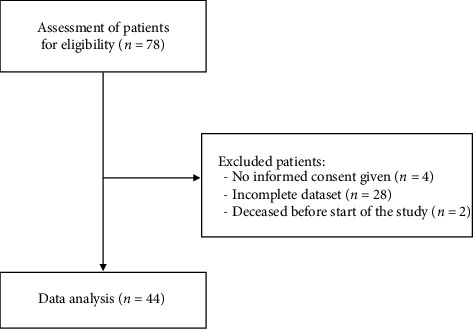
Patient enrollment flowchart.

Seventy-six patients underwent lumbar facet RFA with the 3-tined cannula at our pain center between September 2018 and February 2022 and were evaluated for study eligibility. As illustrated in Figure 3, 44 patients were included in this study. A total of 32 patients were excluded: four did not provide informed consent to the use of their data and 28 patients were excluded due to insufficient data.

Preprocedural information of the patients is summarized in Table 1.

### 3.1. Lumbar Facet RFA Characteristics

The characteristics of lumbar facet RFAs are presented in Table 2. In all patients, multiple facet joint levels were ablated. The L5/S1 facet joint level was the most frequently treated, followed by the L4/L5 and the L3/L4 level.

### 3.2. Primary Outcome Measure

**Figure 4:**
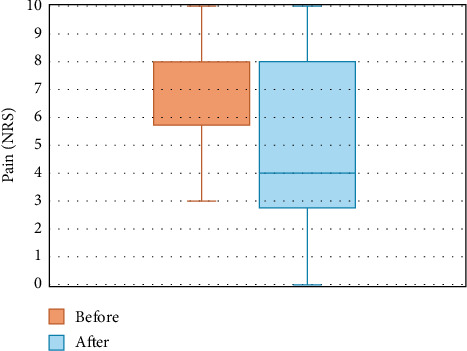
Change in pain intensity from pre- to post-RFA. NRS = numeric rating scale, RFA = radiofrequency ablation.

The mean pain severity at baseline was 7.3 on the NRS (SD = 1.3), which decreased significantly by 2.4 points (95% confidence interval [CI], 1.6–3.2, *p* < 0.001) to a mean NRS value of 4.9 (SD = 3.1) 2 months after the RFA (Figure 4). Out of 44 patients, 23 (52.3%) experienced at least a 30% reduction in pain and 18 (40.9%) experienced at least a 50% reduction at the follow-up. Twenty-five (56.8%) patients had joints bilaterally ablated, resulting in a mean NRS decrease of 1.8 (95% CI, 0.9–2.7, *p* < 0.001), which led to a mean NRS of 5.2 (SD = 3.3) after RFA compared to the initial mean NRS of seven (SD = 1.9). In 19 cases (43.2%), unilateral ablations were performed, with a mean NRS of 7.7 (SD = 1.8) at baseline, decreasing by 3.2 points (95% CI, 1.9–4.5, *p* < 0.001) to a mean of 4.5 (SD = 3.0). In 39 patients (88.6%), two facet joints were ablated, leading to a mean NRS improvement of 2.2 points (95% CI, 1.4–3.0, *p* < 0.001) from a baseline value of 7.3 (SD = 1.9) to a value after the procedure of 5 (SD = 3.1). In one patient, three joints were treated, resulting in an NRS improvement from eight to three. Among the four patients with four ablated joints, the mean NRS at baseline was 7.5 (SD = 1.3), which decreased to a mean NRS of 4.0 (SD = 4.1) after the procedure.

### 3.3. Secondary Outcome Measures

**Figure 5:**
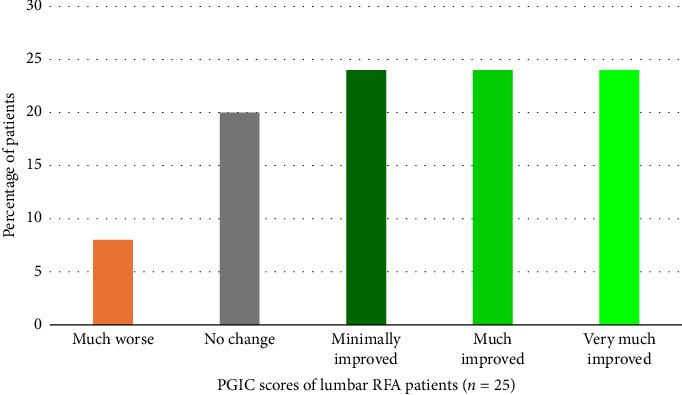
PGIC scores 2 months after RFA in percentage of patients. PGIC = global impression of change, RFA = radiofrequency ablation.

Seventy-two percent of patients reported a PGIC score of at least “minimally improved,” as shown in Figure 5. Forty-eight percent of patients reported a PGIC score of at least “much improved.” Changes in the pain medication use, sleep quality, walking ability, and patient satisfaction are illustrated in Table 3. No patients reported the experience of severe side effects or complications related to RFA treatment. Due to the COVID-19 pandemic, the two-month postprocedural assessments were sometimes conducted by telephone instead of in person. This resulted in more missing values.

## 4. Discussion

We described the novel lumbar facet joint RFA technique with the 3-tined cannula and assessed the prospectively maintained outcomes of a cohort of patients treated with this RFA technique. In this study, lumbar facet joint RFA demonstrated a statistically significant and clinically meaningful pain relief at the 2-month follow-up, with 41% of the patients reporting a greater than 50% pain relief. Almost half of the patients reported a PGIC score of at least “much improved.”

More than half of the patients reduced the use of analgesics, and almost half of the patients who reported on sleep quality and walking ability reported improvement. Out of 25 patients who reported their satisfaction with the result of the treatment, 16 (64%) expressed satisfaction.

Our results align with previously published systematic reviews and meta-analysis [13, 15] on conventional lumbar facet joint RFA. Only one other study has evaluated the efficacy of the lumbar facet joint RFA technique with the 3-tined cannula [26]. The results of this retrospective study, which compared the conventional and the novel technique on separate occasions, demonstrated an even higher pain reduction of four points on the NRS 3 months post-RFA. The findings of a recently published study, evaluating the novel RFA technique with the 3-tined cannula at the cervical facet joints [27], suggest that this technique also results in significant pain reduction and improvement on the PGIC at the cervical level.

The perpendicular approach for lumbar facet RFA closely resembles the approach for the lumbar diagnostic MBBs and is also technically less challenging than the conventional RFA technique. The goal of facet joint RFA is to disrupt the conduction of nociceptive signals along the medial branches from the facet joint to the central nervous system, thereby eliminating the perception of pain by the patient [11]. The medial branches have a small diameter (< 2 mm), and the L5 dorsal ramus diameter is around 0.5 mm [28, 29]. These nerves vary in location in relation to the bony anatomic targets and the rationale for positioning the cannula tip as close to the target nerve and enhancing the lesion size to increase the likelihood of ablating the target nerve [30, 31].

A conventional monopolar cannula (with or without curved active tip) produces a lesion with an elliptical shape along the shaft of the cannula's tip, with only a small cross-sectional footprint at the cannula's tip [32, 33]. Consequently, it is recommended that the conventional RFA cannula is placed parallel to the course of the nerve to improve the likelihood that the RFA lesion envelops the nerve [8, 34]. For conventional RFA of medial branch nerves L1 to L4, not only oblique angulation but also a 15°–25° caudal angulation of the C-arm is required. The RFA cannula is advanced in the groove between the transverse process and the superior articular process and finally positioned parallel to the nerve. For the conventional RFA of the L5 dorsal ramus, a posteroanterior fluoroscopic projection is used, and the target is the junction of the superior articular process of S1 and the upper margin of the ala. The perpendicular technique with the conventional cannula reduces the chance of capturing the nerve [35] due to the aforementioned small cross-sectional lesioning footprint at the cannula tip [32, 33]. Indeed, previous studies concluded that the perpendicular technique with a conventional RFA cannula leads to poorer outcomes compared to the parallel technique [18, 36].

The three tines are deployed from the tip by the manipulation of the handle. The configuration of these tines is pyramid-like, resulting in a 3-dimensional pyramid-shaped lesion with a maximum diameter of approximately 10 mm, in case of a cannula with a 5 mm active tip. The maximum diameter of this larger cross-sectional footprint, located approximately 2 mm above the bony surface, is in close proximity to the medial branch nerve [20]. The tines are flexible and, therefore, able to adapt and conform to the bony surface irregularities upon deployment, reducing the variability of the lesion. We advise against applying pressure to the needle after deployment to increase the lesion area, as this may risk tine breakage.

The larger pyramid-shaped lesion created with the 3-tined cannula decreases the likelihood of missing the target nerve, eliminating the need to reposition the cannula multiple times and conduct numerous lesions. This likely shortens procedure times, potentially reducing radiation exposure and costs [20]. Moreover, the perpendicular technique requires the cannula to pass through less soft tissue in comparison to the conventional parallel technique, leading to less discomfort for the patient.

The perpendicular approach with the 3-tined RF cannula is also likely to be safer than the conventional parallel approach. With the perpendicular approach, the cannula is placed perpendicularly on top of the bony surface. Conversely, with the parallel approach, the cannula is advanced parallel to the nerve without a bony endpoint, increasing the risk of anterior misplacement into the neuroforamen, which places the spinal nerve root at risk of ablation [37]. To ensure diagnostic accuracy, two consecutive positive diagnostic MBBs were required before proceeding with RFA, thereby reducing the risk of the false-positive results [1, 38, 39]. However, we acknowledge the balance between diagnostic accuracy and potential downsides of dual MBBs, including increased patient inconvenience, costs, and risk exposure [17].

Several limitations in this study warrant attention. First, the lack of a control group makes it impossible to compare to other interventions. However, these patients were refractory to conservative treatment and showed a large mean effect size after RFA. The median pain duration in these patients was ≥ 2 years, reducing the likelihood of any influence of natural course confounding the results. Additionally, clinical trials for invasive treatments with control groups face inherent challenges, including securing funding for expensive procedures, difficulty recruiting a sufficient number of patients, and the need for long-term follow-up [40]. Effectively masking and blinding patients to RFA treatments is also difficult, making sham-controlled trials ethically challenging and less feasible. In the study of Deng et al., patients were treated with both conventional and novel RFA technique on different occasions, but patients, clinicians, data collectors, and data analysts were not blinded to the technique [26].

A second limitation was the small sample after the exclusion of participants who did not contribute sufficient follow-up data. This affects statistical power and precision. Nonetheless, in our study, we were still able to demonstrate significant effects on mean NRS scores over time, indicating sufficient power for effect sizes as large as those produced by the treatment.

A third limitation is the fact that no sensory or motor stimulation was performed before facet joint RFA. The omission of sensory and motor stimulation before facet joint RFA is controversial. In our pain management center, we primarily adhere to the International Pain Society and Interventional Spine Society practice guidelines. According to these guidelines, the use of sensory and motor stimulation is not generally recommended as a routine practice before performing RF denervation. Instead, the guidelines emphasize that appropriate cannula positioning is based on anatomic landmarks visualized by an ipsilateral oblique, posteroanterior, and true lateral fluoroscopic view to ensure that the tips are outside of the foramina [11]. However, several other guidelines recommend electrical sensory and/or motor testing to avoid damage to nontargeted spinal nerves or other unintended structures, but the evidence is inconclusive [8, 41]. Additionally, as described above, the perpendicular technique is presumed to be safer than the parallel technique. Therefore, we think that sensory and/or motor testing is less crucial in case of the novel perpendicular RFA technique.

Lastly, our study assessed only a short-term follow-up interval of 2 months. This is due to the retrospective nature of the study and our clinical approach with a follow-up after 2 months for all patients after RF. It should be taken into consideration that the usage of local anesthetics during the RFA procedure itself may have a long-lasting effect on patients with chronic pain. A study demonstrated a relief of more than 50% in pain for about 32 days when applying lidocaine during a MBB [42].

The medial branch nerves regenerate and re-establish facet joint innervation over time [43]. It has been demonstrated that the duration of pain relief after conventional lumbar facet RFA ranges from 6 months to 12 months [13, 44, 45].

## 5. Conclusion

Our study results suggest that lumbar facet joint RFA using the novel lumbar facet joint RFA technique with the 3-tined cannula achieves significant pain relief and improvements on the PGIC. The larger lesion created with the 3-tined cannula decreases the likelihood of missing the target nerve while obviating the need to reposition the cannula multiple times and conduct numerous lesions. Additionally, the perpendicular approach likely leads to shorter procedure times, likely to reduce discomfort, radiation dose, and costs. The novel technique is technically less challenging than the conventional technique, and our results warrant consideration of replacing the conventional technique with the novel technique. However, randomized controlled clinical trials are warranted to confirm the efficacy of the novel lumbar facet joint RFA technique in treating lumbar facet joint pain.

## Figures and Tables

**Table 1:** Baseline characteristics of patients.

Sex (*n* = 44): *n*; %	Female: 21; 47.7
	Male: 23; 52.3

Age (years) (*n* = 44): mean; SD	61.9; 16.8

Duration of pain (*n* = 34): *n*; %	≥ 2 years: 21; 61.8
	≥ 1 year: 6; 17.6
	≥ 6 months: 5; 14.7
	≥ 3 months: 2; 5.9

Baseline pain score (NRS, *n* = 44): mean; SD	7.3; 1.9

Prior lumbar spine surgery (*n* = 44): *n*; %	6; 13.6

Existing posterior spinal instrumentation in at least one ablated level (*n* = 44): *n*; %	1; 2.3

*Note:* Numbers between brackets show the number of available values.

**Table 2:** Lumbar facet RFA characteristics.

Side (*n* = 44): *n*; %	Right: 14; 31.8
	Left: 5; 11.4
	Bilateral: 25; 56.8

Ablations per level (*n* = 68): *n*; %	L2/L3: 7; 7.2
	L3/L4: 8; 8.2
	L4/L5: 34; 35.1
	L5/S1: 48; 49.5

No. of patients per number of ablated facet joints (*n* = 44): *n*; %	2 facet joints: 39; 88.6
	3 facet joints: 1; 2.3
	4 facet joints: 4; 9.1

**Table 3:** Usage of pain medication, sleep quality, walking ability, and patient satisfaction 2 months after lumbar facet RFA treatment.

Usage of pain medication (*n* = 29): *n*; %	Stopped the use: 4; 13.8
	Decreased the use: 12; 41.4
	No change in use: 10; 34.5
	Increased the use: 3; 10.3

Sleep quality (*n* = 29): *n*; %	Improved: 12; 41.4
	No change: 14; 48.3
	Worsened: 3; 10.3

Walking ability (*n* = 27): *n*; %	Improved: 11; 40.7
	No change: 15; 55.6
	Decreased: 1; 3.7

Patient satisfaction (*n* = 25): *n*; %	Satisfied: 16; 64.0
	Not satisfied: 9; 36.0

## Data Availability

The data that support the findings of this study are available from the corresponding author upon reasonable request.
